# Melatonin Use in Pediatric Intensive Care Units: A Single-Center Experience

**DOI:** 10.3390/medsci11030055

**Published:** 2023-08-28

**Authors:** Jessica L. Jacobson, Joanna Tylka, Savannah Glazer, Yanyu Zhang, Rosario Cosme, Jean M. Silvestri, Pallavi P. Patwari

**Affiliations:** 1 Department of Pharmacy, Rush University Medical Center (RUMC), Chicago, IL 60612, USA; jessica_l_jacobson@rush.edu; 2 Department of Pediatrics, RUMC, Rush University Children’s Hospital, Chicago, IL 60612, USA; joanna_c_tylka@rush.edu (J.T.);; 3 Bioinformatics and Biostatistics Core, RUMC, Chicago, IL 60612, USA; 4 Department of Psychiatry, RUMC, Chicago, IL 60612, USA

**Keywords:** melatonin, pediatrics, delirium, sleep, intensive care, circadian, insomnia

## Abstract

Growing evidence indicates that altered melatonin secretion during critical illness may influence the quality and quantity of sleep, delirium, and overall recovery. However, limited data exist regarding the use of melatonin in pediatric critical illness. Data were reviewed over a 5-year period at a tertiary pediatric intensive care unit for pediatric patients (ages 0–18 years) who were prescribed melatonin with the aim of identifying the frequency of and indications for use. Data collection included the hospital day of initiation, the dose, the frequency, the duration of use, and the length of stay. The results demonstrate that melatonin was infrequently prescribed (6.0% of patients admitted; *n* = 182) and that the majority of patients received melatonin as continuation of home medication (46%; *n* = 83 of 182). This group had significantly earlier melatonin use (0.9 ± 2.3 day of hospitalization; *p* < 0.0001) and significantly reduced lengths of stay compared to the other groups (mean LOS 7.2 ± 9.3 days; *p* < 0.0001). Frequently, clear documentation of indication for melatonin use was absent (20%; *n* = 37). In conclusion, given that melatonin is infrequently used within a tertiary PICU with the most common indication as the continuation of home medication, and often without clear documentation for indication, this presents an opportunity to emphasize a more attentive and strategic approach regarding melatonin use in the PICU population.

## 1. Introduction

Melatonin is an endogenous hormone that is synthesized and secreted from the pineal gland with a nocturnal predominance; it plays an essential role in maintaining the body’s circadian rhythm (i.e., the sleep–wake “clock”). Beyond its primary role of regulating the sleep–wake cycle, endogenous melatonin has been reported to be involved in the physiologic maintenance of multiple homeostatic processes, with effects on mood, antioxidative processes, and immunity [1,2,3]. More recently, the exogenous administration of melatonin has been evaluated for use in children as an adjunct for anxiolysis and sedation, with mixed conclusions regarding efficacy [4,5,6,7,8]. The bidirectional role of melatonin secretion and the immune response complicates our understanding of its impact on chronic inflammatory diseases and acute illness [2]. Furthermore, many risk factors for sleep disruption overlap with risk factors for the development of delirium in pediatric intensive care units (PICUs) [9]. Therefore, alterations to normal melatonin secretion during critical illness may play a key role in sleep quality and quantity, delirium, and overall recovery.

PICU patients are at high risk for sleep disruptions due to frequent assessments, invasive monitoring, medication administration (sedation, neuromuscular blockades), reduced or absent nutritional intake, and excessive light and noise levels; this has been recognized for decades [10]. Some investigations support the idea that circadian sleep–wake rhythms are disrupted by fragmented sleep and the absence of diurnal variation during intensive care unit (ICU) admissions [11,12,13,14]. While the underlying disease process can have negative consequences on normal melatonin secretion, this is difficult to evaluate and studies have found no significant differences in melatonin levels based on diagnoses of sepsis, respiratory failure, or neurologic injury [15,16,17], which may be attributable to the pronounced person-to-person variability of endogenous melatonin levels and to the variability in the timing and source of melatonin measurements.

The exogenous administration of melatonin in pediatrics has generally been considered safe for outpatient use, but few studies evaluate the role of exogenous melatonin administration in PICU patients. One study retrospectively reviewed the dose and frequency of melatonin administration in hospitalized pediatric patients, noting a dramatic increase in use (246%) over four years [18], which is in line with the Centers of Disease Control and Prevention’s reports of increased pediatric melatonin ingestion (530%) over a period of eight years [19] suggesting significant increase in outpatient melatonin use in children. 

Studies considering melatonin in adult ICU patients have shown no differences in the occurrence of delirium [20] or the duration of sleep, with one study reporting improvements in subjective sleep quality [21,22]. While sleep and circadian disruption in adult critical illness has been thoroughly explored and is a burgeoning area of research [23], similar work for pediatric critical illness is comparatively sparse (Table 1). One retrospective study evaluated use of melatonin in the PICU for delirium, and the authors found no associated decrease in antipsychotic usage [24]. To our knowledge, this is the first study to provide a descriptive evaluation of how and why melatonin is prescribed in a tertiary PICU. 

## 2. Materials and Methods

This was a single-center retrospective study that was completed in an 18-bed PICU at Rush University Children’s Hospital (RUCH). RUCH is a 110-bed children’s hospital within a tertiary care academic medical center in Chicago, Illinois. Data were collected for a 5-year period from June 2015 through June 2020, which was divided into a baseline period (3.05 years) and a post-delirium-education period (1.98 years). Patients in both timeframes were identified from an electronic medical record (EMR)-generated list of medication orders for melatonin. All of the administered melatonin was dispensed by the pharmacy on formulary. Patient-specific data collected included age at hospitalization, length of stay (LOS), and reason for hospital admission (classified as either a surgical admission or medical admission). Data collected that were specific to melatonin use included the melatonin dose and frequency of administration, the duration of inpatient therapy, the hospital day of initiation, and continuation at discharge. If melatonin was ordered to be given more than once (every 24 h) during a patient’s admission, this was classified as “nightly” and distinguished from the administration of a single dose. The indication of melatonin use during hospital admission was based on chart review and classified as one of the following: (1) delirium-related sleep disturbance, (2) insomnia, (3) circadian disorder such as a “flipped” sleep–wake schedule, (4) continuation of home medication, or (5) “unknown” when the indication was not clearly documented in the medical chart.

As a quality improvement (QI) initiative, pediatric delirium education, the implementation of routine delirium screening, and a treatment algorithm were developed by a multidisciplinary team including pediatric intensivists, a child psychiatrist, a pediatric clinical pharmacist, and a PICU nurse/acute care pediatric nurse practitioner student. The delirium identification and documentation processes used the Cornell Assessment of Pediatric Delirium (CAPD) [26] tool on paper, and later routine scoring was documented in the EMR. The clinical pathway used to standardize the care of patients with delirium in the PICU was simplified from the American Academy of Pediatrics’ publication from 2019 [27]. All PICU patients with a hospital stay ≥24 h received non-pharmacologic preventive interventions and CAPD screening every 12 h. For patients with positive CAPD scores (≥9), interventions included: (1) discussing the weaning of benzodiazepines and increasing/adding dexmedetomidine or clonidine for sedation, when possible, (2) considering melatonin if sleep disturbance was present, and, if persistently elevated CAPD scores were recorded, then (3) considering the initiation of antipsychotic medication with possible pediatric psychiatry consultation. Melatonin was recommended in our pathway for patients with sleep disturbance based on clinical evaluations, since this information cannot be identified from the CAPD score. The melatonin enteral dosing recommendations suggested giving melatonin nightly at 1.5 mg for infants, 3 mg for children, and 5 mg for adolescents (while acknowledging that there is an absence of clear dosing recommendations for melatonin). 

The primary aim of this study was to explore the practices of prescribing melatonin in the PICU, with variables including the indication, dose, timing of initiation compared to LOS, duration of treatment, and frequency of administration. The secondary outcome was to evaluate changes in melatonin prescribing practices following unit-based delirium education. 

For the statistical analysis, categorical variables were presented as percentage frequencies, with continuous variables presented as means ± standard deviations. Categorical variables were analyzed using the Chi-square test or the Fisher exact test, as appropriate. Continuous variables were analyzed using the Mann–Whitney U test or Kruskal–Wallis test, as appropriate. Post hoc analysis was performed with Dunn’s test on the indication of melatonin administration with the Bonferroni correction. Statistical significance was defined as *p* < 0.05. Analyses were performed using SAS v9.4 (SAS, Cary, NC, USA) and R Studio software. 

## 3. Results

A total of 182 patients (6.0% of total PICU admissions over the five-year period) received melatonin and were included in the study. The majority of patients (*n* = 153; 84%) were admitted for a medical (non-surgical) issue and 16% (*n* = 29) had a diagnosis of sepsis. The median LOS was 7.0 days (mean 25 ± 56 days) with 18% (*n* = 33) hospitalized for >30 days and 5% (*n* = 9) of patients having a LOS > 90 days. There were no significant differences in terms of admission classification, sepsis diagnosis, or LOS between the baseline group and the post-delirium-education group (Table 2). 

There was wide variability in the timing of the initial melatonin dose (average 10.1 ± 31.2 days). Excluding those who began receiving melatonin on day 0 as a home medication, the median hospital day of initial melatonin administration was day 4. Melatonin was initiated early in the admission for 38.5% of patients on the day of admission (hospital day 0) and for 54.9% of patients on hospital day 0 or 1. Almost one-third of patients (*n* = 58) continued taking melatonin as a home medication. Patients received melatonin on average for 9.9 ± 25.6 treatment days, equivalent to 63% ± 35% of the duration of their hospitalization. The average dose of melatonin prescribed was 4.0 ± 2.3 mg (range: 1.0 to 20.25 mg; five patients received melatonin ≥ 10 mg) and the majority of patients (93%) received nightly scheduled melatonin (every 24 h), rather than one dose for a single night. Melatonin was prescribed at discharge for 115 (64%) patients; 3 patients were excluded (1 death and 2 cases for which the prescription of melatonin at discharge was not clear in the medical documentation). Of those discharged with melatonin, 45% (*n* = 80 of 179) received melatonin during hospitalization as a continuation of home medication (Table 3).

The frequency of melatonin administration within our PICU nearly doubled following delirium education (8.3% of admissions, 53 cases per year) compared to the baseline group (4.4% of admissions, 26 cases per year). No statistically significant differences were found in patient characteristics or melatonin administration practices between the baseline and post-delirium-education periods (Table 1). 

**Figure 1:**
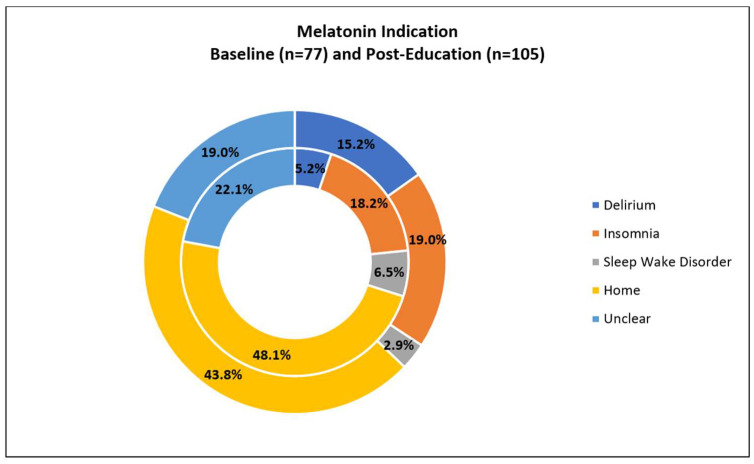
Indications for melatonin administration when comparing the baseline and the period after the implementation of pediatric delirium education and screening. The initial (baseline) group is represented by the inner ring (4.4% of total PICU admissions over three years). The post-delirium-education group is represented by the outer ring (8.3% of total PICU admissions over two years). Note that the most common indication for both groups is continuation as a home medication. Melatonin given for delirium nearly triples when comparing the baseline group to the post-delirium-education group.

**Figure 2:**
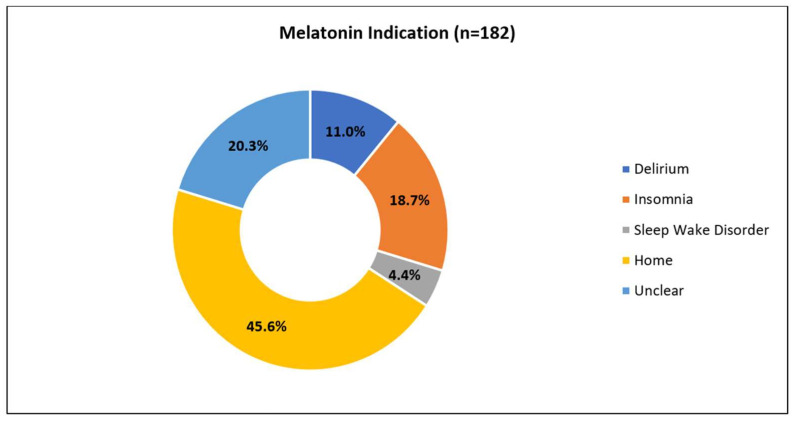
Indications for melatonin administration for the total study population. The most common indications for melatonin use in this PICU cohort are the continuation of home medication followed by unknown (unclear indication based on chart review).

The most common indication for melatonin use during hospitalization was the continuation of a home medication (45.6%, *n* = 83 of 182; 2.7% of all PICU admissions) and the least common was a diagnosis of a circadian sleep–wake cycle disorder (4.4%, *n* = 8). The indication for melatonin administration was not clearly documented (indication “unknown”) for 20.3% (*n* = 37) of patients; it was recorded as insomnia for 18.7% (*n* = 34) and delirium for 11.0% (*n* = 20) (Figure 1). The use of melatonin for delirium-related sleep disturbance increased from 5.2% in our baseline group to 15.2% in our post-education group (with the isolated evaluation of delirium *p* = 0.032) (Figure 2). 

In our cohort, melatonin was not given for anxiolysis, as an adjunct to sedation, or as an anti-inflammatory agent. The median dose of melatonin for all indications was 3 mg. When comparing indications for melatonin use, there were statistically significant differences in the LOS, day of melatonin initiation, total treatment days per LOS, and frequency of melatonin prescribed at discharge (*p*-value < 0.0001) (Table 4).

In the post hoc analysis, differences were found for patients receiving melatonin as a continuation of home medication compared to all other groups—these patients were started on melatonin sooner (average 0.9 ± 2.3 hospital day), received melatonin for a greater percentage of their hospitalization (average 85% ± 27%), and were more frequently prescribed melatonin upon discharge (98%). In comparing the LOS, patients using melatonin as a home medication had a shorter length of hospital stay (7.2 ± 9.3 days; median 4 days), which was significantly shorter than the indications for delirium-related sleep disturbance (*p* < 0.0001), insomnia (*p* = 0.0003), sleep–wake disorder (*p* = 0.001), and “unknown” indications (*p* = 0.0164). 

Patients receiving melatonin as a treatment for delirium-related sleep disturbance had the longest length of stay of 92.2 ± 136.5 days (median 39 days), with melatonin being started at a later hospital day (46.2 ± 78.8 days; median 12.5 days) compared to the other indications. Only one patient with delirium was started on melatonin on hospital day one. In total, 65% (*n* = 13) of patients who were on melatonin for delirium-related sleep disturbances were prescribed melatonin at discharge. 

An additional analysis was completed that excluded those patients with a melatonin indication of “continuation of home medication” (*n* = 83 excluded). As expected, melatonin was initiated at a slightly later day of hospitalization (day 17.9 ± 40.7) and for a shorter duration of the patients’ hospitalizations (44% ± 30%). In this subset of patients, no differences were found in terms of patient characteristics in the baseline group and the post-delirium-education group. 

## 4. Discussion

To our knowledge, this is the first study to closely evaluate current practices including indications of exogenous melatonin administration for patients in the PICU. While several therapeutic roles have been proposed for melatonin use beyond facilitating sleep onset, including anxiolysis (pediatric pre-operative use) [4,5,6,7,8], antioxidative and autoimmune properties (neonatal asphyxia [28,29] and sepsis [30]), and for outpatient neurodevelopmental disorders [31,32], the potential indications and benefits of exogenous melatonin administration in children who are hospitalized with critical illness have not been fully established. 

The proposed benefits of melatonin use in hospitalized children with critical illnesses include protection against ICU delirium through the maintenance of normal sleep patterns and sleep durations during hospital admission to promote rapid recovery from acute illness. In addition, melatonin administration has been suggested to play an adjunctive role to sedation and analgesia, decreasing nocturnal blood pressure, and decreasing nocturnal seizures in PICU patients [33]. In adult ICU patients, conflicting literature exists, as some studies have failed to show improvements in sleep duration and sleep quality with the use of melatonin and melatonin receptor agonists in adult patients [22,34,35], while one study did find improved sleep quality [21]. As for delirium prevention, a recent meta-analysis found no evidence of a reduction in delirium with melatonin or ramelteon administration in adult ICU patients when considering nine randomized controlled studies. However, it did note a reduction in the risk of delirium when including four other studies that did not fully meet the inclusion criteria [20]. Differences in patient populations, melatonin doses, and primary efficacy endpoints, as well as overall small sample sizes, make it difficult to draw conclusions about the efficacy of melatonin use in the ICU population. With even fewer studies on the PICU population, the benefits of melatonin use in the PICU population remain uncertain. 

In our study, melatonin was infrequently prescribed in our PICU (6% of all admissions) over the five-year study period. The low overall usage may have been due to a multitude of factors, including a lack of studies demonstrating the proposed benefits of melatonin, unclear dosing guidelines in infants and children, concern for potential drug–drug interactions, unclear side effects, and/or the dismissal of the medication’s effect based on its categorization as an herbal product. Proposed melatonin dosages for therapeutic effects in adults range from 0.5–20 mg/dose; however, the ideal pediatric dose has not been established. A randomized, placebo-controlled double-blind trial in children 6–12 years of age with chronic sleep-onset insomnia found melatonin to be effective in advancing sleep onset (falling asleep earlier in the evening) and reducing sleep onset latency (time to fall asleep) at a dose of 0.05 mg/kg when given at least 1 to 2 h before the desired bedtime. However, no dose–response relationship was seen within a dose range of 0.05–0.15 mg/kg/dose (equivalent to 0.5 to 1.5 mg for the average toddler, and 1.6 to 4.8 mg for the average 10-year-old); rather, a significant treatment effect was found based on the timing of the melatonin administration [36]. Further complicating melatonin dosing is its status as a dietary supplement, with a lack of regulation of its formulation potency and purity resulting in variability based on the product and dosage [37]. 

With an oral intake of 10 mg of melatonin in an adult population (*n* = 12), the time to reach maximum plasma concentration was 40.8 ± 17.8 min with a median absolute bioavailability of 2.5% (1.7–4.7%). The maximum peak serum concentration and area under the curve showed extensive inter-individual variability [38]. Pharmacokinetic data in children are currently unavailable. Melatonin is metabolized primarily through CYP1A2 metabolic pathways and has the potential for drug–drug interactions with other medications that are metabolized through the same cytochrome P450 systems [39]. 

The adverse effects appear to be minor and include headaches, drowsiness, dizziness, stomach cramps, irritability, and diarrhea [40]. One systematic review of the outpatient use of melatonin for pediatric chronic insomnia found no serious adverse events after the short- or long-term administration of melatonin to children and adolescents, but the authors did note increased non-serious events such as headaches, nausea, behavior changes, or fatigue/drowsiness [41]. Furthermore, these non-serious adverse events were not affected by patient age and gender or by the melatonin duration or dose [41]. Another systematic review of the pre-operative use of melatonin (as an anxiolytic) also demonstrated an “excellent safety profile” [6]. Despite a lack of large clinical trials, a growing number of studies report the safe use of melatonin in the pediatric population [42]. In our study, the evaluation of adverse effects of melatonin could not be easily discerned. 

Melatonin use as a continuation of a home medication was found in 2.7% of our study population, which is higher than the reported 0.1–0.7% use of melatonin dietary supplements in the US for 4–17 year old children from 2007 to 2012 [43]. The dramatic increase in reported melatonin ingestions (0.6% in 2012 and 4.9% in 2021) suggests that the use of melatonin in the US has also increased since 2012 [19], which may be due to the increased prevalence of insomnia and an increase in availability of child-friendly products (in chewable, gummy, or liquid forms). Additionally, it is unclear whether there has been a shift in the outpatient use of melatonin for healthy children compared to those with chronic and complex medical conditions who may have more frequent PICU admissions. Furthermore, unavailable clinical documentation made it difficult to ascertain whether the at-home melatonin use seen in our population was secondary to self-prescribing or pursuant to a provider’s recommendation. This study also found significant differences based on the indications for melatonin initiation. Patients prescribed melatonin in the PICU for indication as a home medication were started on melatonin earlier in their hospital course, had a longer duration of treatment as a percentage of their total LOS, and were more frequently prescribed melatonin at discharge as compared to other indications for initiation. The hospital LOS was significantly shorter in patients who continued to receive melatonin as a home medication. These findings are not surprising, but the implications that melatonin is a preventive/protective medication that assists with recovery time remain uncertain. 

Our study also showed increased comfort with prescribing melatonin after the initiation of delirium education, with 4.4% vs. 8.3% of PICU admissions receiving melatonin therapy pre- and post-intervention, respectively. Although not statistically significant, a younger age at the time of melatonin initiation was seen in the post-delirium-education group (8.6 ± 5.9 years) compared to the baseline group (10.3 ± 5.6 years). This suggests that challenges to using melatonin in the PICU could be overcome through more focused education, including improved awareness regarding the close relationship between sleep disruption and delirium. There are currently no national guidelines for the treatment of delirium in the PICU and the current recommendations are based on adult data and small pediatric studies [44]. The prevalence of pediatric delirium being 20–44% in PICUs nationally and 24% at our PICU (data not shown) requires providers to have a more standardized approach that is based on both evidence-based practice and expert consensus. We found that implementation of delirium education and the implementation of routine delirium screening with a delirium prevention and treatment algorithm resulted in a higher use of melatonin, specifically for delirium-related sleep disturbances, ranging from 5.2% to 15.2% (Figure 2). The increased use of melatonin following the implementation of our quality initiative was anticipated due to it facilitating a consistent approach for the recognition of delirium-related sleep disturbances in the pediatric population and because the algorithm has initial steps for medication modification prior to the initiation of antipsychotic agents. 

Interestingly, the highest LOS was found in the group of patients who received melatonin for delirium-related sleep disturbances. This may be explained by the fact that patients with prolonged hospitalizations are at higher risk of developing delirium. In general, it may be that there is higher medical complexity (i.e., having other risk factors for delirium) in the patients who received melatonin for delirium-related sleep disturbances. One recent study that considered using melatonin to treat delirium in a PICU found that the initiation of melatonin was associated with decreased sedation scores, and, while not statically significant, it was also associated with decreased days of sedation and pain scores [24]. Further research is needed to clarify the differences in responses to melatonin when given as preventative/maintenance medication rather than being used to treat sleep disruption and delirium after diagnosis. 

The results of this study are limited by a few factors. First, our study is a single-center study, which limits the total number of patients included and offers less variability in providers (and their associated practice-based differences). Due to the retrospective nature of this study, there were some inconsistencies in the EMR documentation practices for melatonin indications and reasons for discontinuation. This ultimately left a large subset of patients (20%, *n* = 37) with an unknown indication for melatonin, leaving a gap in our understanding of why melatonin was prescribed. While this may reflect the general perception that melatonin administration is benign or inconsequential, these findings reinforce the importance of encouraging providers to treat melatonin as a “medication”, with clear documentation for the indication of use. In addition, our study is limited by an absence of distinguishing clinical features amongst the cohorts (melatonin indications) evaluated. Therefore, the differences in the LOS among the melatonin indication groups could be due to differences in illness severity or underlying chronic conditions among the groups that could not be evaluated based on retrospective data collection. Lastly, our descriptive study did not evaluate efficacy and safety endpoints related to melatonin use in critically ill children. Further studies are needed to evaluate the impact of melatonin on sleep quality/duration and delirium prevention and to evaluate the potential benefits of melatonin in relation to LOS.

## 5. Conclusions

This study describes how melatonin is being prescribed in a tertiary PICU, with an overall low percentage of patients prescribed melatonin; it was most frequently given as continuation of home medication (associated with earlier administration and shorter LOS). The second most common indication for melatonin administration was “unknown”, suggesting that providers need to be more attentive to indications for melatonin use, as they would for any other medication. Increased melatonin administration following the implementation of delirium education, routine screening, and a clinical treatment pathway could be attributed to the improved recognition of pediatric-delirium-related sleep disturbances and/or greater comfort levels with prescribing melatonin. These findings also emphasize the need for a more thoroughly scrutinized and strategic approach regarding melatonin use in the PICU population and the need for further research.

## Figures and Tables

**Table 1:** Studies reporting melatonin levels or use in PICU patients.

Melatonin	Study Type	*n*	Publication Year	Author
Exogenous	Retrospective	63	2021	Laudone [24]
Exogenous	Retrospective	# (379)	2021	Procaccini [18]
Endogenous	Prospective, observational	50	2020	Foster [17]
Endogenous	Prospective, observational	24	2017	Marseglia [25]
Endogenous	Prospective	16	2013	Marseglia [14]
Endogenous	Observational	20	2012	Bagci [16]
Endogenous	Observational	36	2011	Bagci [15]

Abbreviations: pediatric intensive care unit (PICU). # Value calculated from reference to ICU-only patients and includes PICUs, cardiac PICUs, and NICUs.

**Table 2:** Cohort characteristics.

Characteristic	Total	Baseline	Post-Delirium Education	*p*-Value
Timespan of chart review (years)	5.0	3.05	1.98	---
Total number of patients	182	77	105	---
Percentage of total PICU admissions	6.0%	4.4%	8.3%	<0.0001 †
Age (years): mean (median), Range, IQR	9.3 (10.6)	10.3 (11.2)	8.6 (7.6)	0.0671 #
	0.3–18.4	0.3–18.4	0.3–18.3	
	3.8–14.3	4.9–15	3.1–13.8	
Admission classification: medical, *n* (%) surgical, *n* (%)	153 (84%) 29 (16%)	64 (83%) 13 (17%)	89 (85%) 16 (15%)	0.765 †
Sepsis diagnosis, *n* (%)	29 (16%)	9 (12%)	20 (19%)	0.180 †
LOS (days): mean (median), IQR	25.0 (7.0) 3–22	27.3 (7.0) 3–26	23.21 (8.0) 3–20	0.688 #
LOS > 30 days, *n* (%)	33 (18%)	17 (22%)	16 (15%)	0.237 †

Abbreviations: interquartile range (IQR), length of stay (LOS), standard deviation (SD). Statistical test: Chi-Square test (†), Mann–Whitney U test (**#**).

**Table 3:** Characteristics of melatonin administration.

MELATONIN	Total	Baseline	Post-Delirium Education	*p*-Value
Dose (mg), (median), mean ± SD, IQR	(3.0), 4.0 ± 2.3 mg 3–5	(3.0), 4.4 ± 2.8 mg 3–6	(3.0), 3.8 ± 1.8 mg 3–5	0.335 #
Frequency of administration Nightly (every 24 h), *n* (%) single night, *n* (%)	170 (93%) 12 (7%)	70 (91%) 7 (9%)	100 (95%) 5 (5%)	0.245 †
Hospital day of initiation (day), (median), mean ± SD, IQR	(1.0), 10.1 ± 31.2 0–7	(1.0), 10.5 ± 30.2 0–5	(1.0), 9.8 ± 32 0–7	0.516 #
* Hospital day of initiation (day), * excluding day 0 and home medication (median), mean ± SD, IQR	*n* = 124 (4.0), 14.9 ± 36.9 1–11.5	*n* = 49 (3.0), 16.7 ± 36.7 1–14	*n* = 75 (5.0), 13.8 ± 37.2 1–11	0.833 #
Total days of administration, (median), mean ± SD, IQR	(4.0), 9.9 ± 25.6 2–9	(4.0), 11.2 ± 35.0 2–7	(4.0), 8.9 ± 15.7 2–9	0.690 #
Treatment days per LOS (%), (median), mean ± SD, IQR	(67%), 63% ± 35% 29–100%	(67%), 63% ± 37% 24–100%	(67%), 62% ± 34% 33–100%	0.849 #
* Treatment days per LOS (%), * excluding day 0 and home medication (median), mean ± SD, IQR	*n* = 124 (50%), 47% ± 31% 18–72%	*n* = 49 (46%), 45% ± 33% 17–71%	*n* = 75 (50%), 48% ± 30% 20–73%	0.553 #
Continuation at discharge, *n* (%)	115 of 179 (64%)	50 of 76 (66%)	65 of 103 (63%)	0.711 †

* Excluding cases with melatonin started on day 0 of hospitalization and reported as a home medication. Abbreviations: interquartile range (IQR), length of stay (LOS), standard deviation (SD). Statistical test: Chi-Square test (†), Mann–Whitney U test (**#**).

**Table 4:** Indication for melatonin administration at a tertiary care PICU over five years.

Value	Delirium (*n* = 20)	Insomnia (*n* = 34)	SWD (*n* = 8)	Home (*n* = 83)	Unknown (*n* = 37)	*p*-Value
Age (years), (median), range, IQR	(6.9), 0.3–17.7 0.9–14.4	(9.4), 0.3–18.3 2.9–15.3	(6.5), 0.3–17.4 3–14.1	(11.4), 0.7–18.4 5.3–13.7	(9.4), 0.3–17.7 3.1–14.1	0.372
Age (years), mean ± SD	7.6 ± 6.9	9.1 ± 6.5	8.1 ± 6.4	10.2 ± 5	8.7 ± 6.2	
Admission classification Surgical, *n* (%) Medical, *n* (%)	4 (20%) 16 (80%)	10 (29%) 24 (71%)	1 (13%) 7 (88%)	11 (13%) 72 (87%)	3 (8%) 34 (92%)	0.133 †
Nightly (Q24h), *n* (%)	20 (100%)	29 (85%)	8 (100%)	80 (96%)	33 (89%)	0.117 #
Dose (mg), (median), range, IQR	(3.0) 1.0–5.0 3–3.5	(3.0), 1.0–7.5 3–5	(3.0), 1.5–6.0 3–3.8	(3.0), 1.0–20.25 3–6	(3.0), 1.0–12.0 3–3	0.014 ⁱ
Dose (mg), mean ± SD	3.2 ± 1	3.7 ± 1.5	3.4 ± 1.3	4.7 ± 2.8	3.6 ± 1.8	
LOS (days), (median), mean ± SD, IQR	(39.0), 92.2 ± 137 16.5–90.5	(12.0), 28.1 ± 37.3 5–27	(26.0) 37.4 ± 29.4 15.5–66	(4.0) 7.2 ± 9.3 2–8	(9.0) 22.9 ± 34.5 3–29	<0.0001 ⁱ
Melatonin started (hospital day), (median), mean ± SD, IQR	(12.5) 46.2 ± 78.8 7–30.5	(3.5) 11.4 ± 18.6 1–12	(14.0) 13.3 ± 9.8 7.5–15	(0.0) 0.9 ± 2.3 0–1	(3.0) 9.5 ± 18.5 1–8	<0.0001 ⁱ
Melatonin Duration (days), (median), mean ± SD, IQR	(12.0) 37.1 ± 68.3 5–34	(3.5) 6.8 ± 8 2–10	(12.0) 17.6 ± 19.9 6–23.5	(3.0) 5.9 ± 8.7 1–7	(3.0) 5.2 ± 6.4 2–6	0.0001 ⁱ
Treatment days per LOS (%), (median), mean ± SD, IQR	(53%) 44 ± 26% 15–65%	(33%) 42 ± 32% 14–67%	(45%) 42 ± 19% 23–55%	(100%) 85 ± 27% 75–100%	(44%) 45 ± 33% 20–65%	<0.0001 †
Melatonin prescribed at discharge, *n* (%)	13 (65%)	14 (42.4%)	3 (37.5%)	80 (97.6%)	5 (13.9%)	<0.0001

Abbreviations: interquartile range (IQR), length of stay (LOS), every 24 h (Q24h), standard deviation (SD), sleep wake disorder (SWD). Statistical tests: Chi-Square Test (†), Kruskal–Wallis Test (**^i^**), Fisher’s Exact Test (**#**).

## Data Availability

The data presented in this study are available on request from the corresponding author. The data are not publicly available due to privacy restrictions.
